# Comparison of Bone Turnover Markers between Young Adult Male Smokers and Nonsmokers

**DOI:** 10.7759/cureus.6782

**Published:** 2020-01-27

**Authors:** Ahmad M Al-Bashaireh, Ola Alqudah

**Affiliations:** 1 Faculty of Nursing, Al-Ahliyya Amman University, Amman, JOR; 2 Family Medicine, King Fahad Medical City (KFMC), Riyadh, SAU

**Keywords:** smoking, tobacco, bone, bone turnover, bone resorption, lifestyle, physical activity, alcohol intake, young adults, osteoporosis

## Abstract

Background

This study aims to compare the differences in the means of bone formation and resorption markers between young adult male smokers and nonsmokers.

Methods

This study employed a cross-sectional, descriptive design. Thirty-five smokers and 38 nonsmokers were recruited. All participants completed self-reported questionnaires about demographics, physical activity, and smoking status. In addition, blood specimens were collected to determine serum levels of bone turnover markers.

Results

Regarding bone formation markers, the least square means (LSM) for osteoprotegerin (OPG) and procollagen type I N-terminal propeptide (PINP) were similar for smoking and nonsmoking groups. Regarding bone resorption markers, the LSM serum carboxyl-terminal telopeptide of collagen type I (CTXI) level was found to be significantly lower in smokers than nonsmokers [0.82 ± 0.83 vs. 1.30 ± 0.82 ng/mL, F (1, 66) = 5.73, p = 0.020]. The LSM for soluble-receptor activator of nuclear factor-kappa B ligand (sRANKL) [1.64 ± 0.60 vs. 1.69 ± 0.62 ng/mL, F (1,64) = 10.74, p = 0.002] and RANKL/OPG [2.62 ± 1.09 vs. 2.81 ± 1.10 ng/mL, F (1,65) = 5.88, p = 0.018] were different for smoking and nonsmoking groups. Exploration of the moderating influence of physical activity on smoking effects revealed significant effect for the interaction between smoking status and physical activity on sRANKL [F (2, 64) = 8.63, p = 0.001] and RANKL/OPG ratio [F (2, 65) = 5.49, p = 0.006].

Conclusion

Our study provides evidence for the effect of smoking on bone resorption markers in young adult males. Such effects should be carefully considered side by side with other lifestyles that may lead to poor bone health and increased risk for osteoporosis.

## Introduction

The metabolism of adult bony tissue is characterized by continuous coupled processes of bone formation and resorption which is necessary to maintain its normal structure and functions [1]. Once there are imbalances in those processes, the mass and the microarchitectural structure of bone are affected. Osteopenia, osteoporosis, and fractures are examples of bone disorders that result from the poor structure and reduced density. Osteopenia is a condition that acts as an early indicator for a decrease in bone mass; it is asymptomatic, but if not managed early may deteriorate to osteoporosis [1]. Osteoporosis is a common disorder of skeletal system that is characterized by reduced bone mineral density (BMD) which makes the bone more fragile and vulnerable to fractures [2]. Fracture is another common bone problem that may occur independently or along with osteoporosis. In the United States, by the year 2025, it is expected that three million fractures will occur annually because of osteoporosis, with a direct medical cost of $25.3 billion [3].

In the United States, according to the 2015 report, the prevalence rate of current smoking in all adults was 15.1% and it was 16.7% in adult males [4]. Active and passive smoking was causally linked with 500,000 annual deaths in the United States [4,5]. Tobacco smoke has thousands of harmful compounds, nicotine and many other compounds such as polycyclic aryl hydrocarbon are examples of these compounds which has a great influence on bone metabolism [5,6]. Disorders of reduced bone mineral density, particularly osteoporosis, is well-known to be related to several factors such as age, genetics, hormonal, and nutritional factors [2]. Smoking is another modifiable risk factor for these disorders and was counted in the Fracture Risk Assessment Tool [7]. Recent evidence determines that tobacco smoke causes an imbalance in bone turnover mechanisms, thus leading to decreases in BMD that makes the bone more vulnerable to osteoporosis and fracture [6,8-10]. Moreover, the current report of Surgeon General was causally linked tobacco smoking with hip fracture and periodontitis [5]. Regarding research conducted in males, cross-sectional and cohort studies found smokers had low BMD than that of the nonsmokers [11-14]. Despite the evidence supporting the adverse effects of smoking on bone health, the mechanism of how smoking induces these changes in bone turnover and reduces bone mass are not fully understood [6,15]. The current advances in bone turnover markers may help to understand the effect of smoking on bone health.

The earlier studies in this field focused on the relationships between smoking and bone mass represented by BMD. However, recent studies explore the relationship between smoking and bone turnover represented by bone turnover markers. The superiority of bone turnover markers over the diagnostic imaging tools lies in its ability to provide a holistic picture for the whole human skeleton and its ability to catch the earliest changes in bone metabolism [16]. Furthermore, nowadays, bone turnover markers are used as a complementary tool for BMD to: (1) provide better understanding for the dynamic of bone turnover in bone metabolic disorders, (2) predict risk of osteoporosis earlier, (3) evaluate the response toward the therapies, and (4) predict the risk of fracture earlier and independent of the BMD [16,17].

Over the last decade, few studies have investigated the relationships between smoking and bone turnover markers, with most of these studies conducted in a lab or individuals suffering from periodontitis rather than in a healthy population. Animal studies found that male rats who were exposed to smoke had significantly higher levels of Tartrate Resistant Acid Phosphatase Isoenzyme-5b (TRACP-5b), and lower bone-Alkaline Phosphatase (b-ALP) activity levels than the controls [18]. In another study, rats exposed to nicotine added to their water had significantly higher levels of osteoprotegerin (OPG) than rats who were not exposed to nicotine [19]. Regarding human studies, there were few studies comparing levels of soluble-receptor activator of nuclear factor-kappa B ligand (sRANKL), OPG, and RANKL/OPG ratio between smokers and nonsmokers. sRANKL and RANKL/OPG ratio are markers for bone resorption, and higher levels are associated with bone thinning. OPG is a marker of bone formation, and higher levels are correlated with increased BMD. In these studies, three reported that smokers had significantly lower levels of OPG [20-22], two reported smokers had a significantly higher level of RANKL/OPG ratio [21,22], and no studies reported that RANKL level significantly differed between smokers and nonsmokers [20-23]. In two studies examined levels of other bone turnover markers: one study reported smokers had significantly lower levels of carboxyl-terminal telopeptide of collagen type I (CTXI) [20], and another study reported smokers had significantly lower levels of osteocalcin (OC) [24].

With the scarcity of empirical knowledge about the effect of tobacco smoking on bone turnover markers, there is a substantial need for studies in this field to understand such an effect. Thus, our study aims to compare the differences in the means for bone formation (OPG, and procollagen type I N-terminal propeptide [PINP]) and resorption markers (sRANKL, RANKL/OPG ratio, and CTXI) between two groups of smokers and nonsmokers of the young adult male population.

## Materials and methods

Study design and setting

Our study employed a cross-sectional, descriptive design aimed to study differences in the serum levels of bone turnover markers between young adult male smokers and nonsmokers. All eligible participants were recruited in a period between September and December of 2018 from Nurse-managed Ambulatory Care Clinic located in Florida. This clinic provides a high quality of health care services for children, adults, and families, accepts most of the insurance plan, and offers reduced fees for uninsured patients.

Ethics statement, recruitment, and data collection

Our study was conducted in harmony with the Declaration of Helsinki and was approved by the University of Florida Institutional Review Board (IRB201701277). Passive techniques (flyers and brochures) were used to recruit eligible participants. Flyers and brochures were posted in the waiting area and clinics of Nurse Practitioners. Before enrollment, potential participants were asked for their readiness to join our study and were screened for eligibility, and if criteria were met, they were asked to sign a consent form. Inclusion criteria were: young adult (20-45 years), fluent in reading and writing English, exhaled carbon monoxide (EXCO) less than 6 ppm for the nonsmoker (never smoker) and more than or equal to 6 ppm for smokers, and smokers need to have regularly smoked over the last three months, currently called “Everyday smoker”. Exclusion criteria were any person diagnosed with vitamin D deficiency, high or low bone turnover diseases, dissociation between bone formation and resorption, chronic diseases with limited mobility, rheumatoid arthritis, chronic obstructive pulmonary disease (COPD), GI diseases (Crohn's and ulcerative colitis), and chronic renal or liver disorders. Also, individuals on medication affecting bone metabolism [e.g., vitamin D, calcium, anti-resorptive, glucocorticoids, aromatase inhibitors, antiepileptic, heparin, thiazolidinedione, vitamin K antagonist, and hormones affect bone metabolism (e.g., testosterone, thyroxine)] were excluded [16,17]. Out of the 84 participants who were screened, 73 met eligibility criteria. Reasons for the 11 excluded were thyroid disorders (n = 2), recent fracture (n = 1), depression (n = 2), rheumatoid arthritis (n = 1), COPD (n = 2), renal impairment (n = 1), use of corticosteroid (n = 1), and smoking of cannabis (n = 1).

Measures

Demographics

A self-reported questionnaire was used to collect participant’s data about age, race/ethnicity, marital status, level of education, living environment, household income, and work status. Also, body composition data were collected, including weight, height, and any significant variations in body weight during the last three months. Body mass index (BMI) was calculated from the participant’s weight and height.

Physical Activity

Physical activity was measured by two items: the number of days over the last week that participant performed any physical activities (>30 minutes), and the number of days over the last week that participant performed any weight-bearing exercise. Responses for items under physical activity could range between 0 and 7.

Nutrition

Nutritional information was assessed by asking participants for (1) use of any vitamins/minerals supplements, and (2) number of days over the last week that participant has consumed any milk/dairy products; responses for this item ranged between 0 and 7. Participants were also asked about their average consumption of alcohol (glass/day).

Smoking Status/History

Smoking status was assessed via self-reported questionnaires which included items about participant smoking status, duration of smoking, the primary mode of smoking, number of cigarettes/puffs per day and week, and detailed assessment regarding the exposure to indoor secondhand smoking.

Exhaled Carbon Monoxide (EXCO)

EXCO was measured using a simple non-invasive commercial device (Micro+ basic Smokerlyzer, Bedfont Scientific, Harrietsham, UK). The detection range for EXCO in this device was 0-150 ppm. Manufacturer instructions were followed during the measurement: Patients take a deep breath and hold for 15 seconds, then exhale within the device, which displays EXCO level on the device screen. The level of EXCO was used in conjunction with the self-reported questionnaire to determine the smoking status of the participant. Participants with 6 ppm or greater were considered as smokers.

Serum Cotinine

Cotinine acts as an exposure marker for tobacco smoking; it is a derivative of nicotine. Cotinine is more stable than nicotine, and it has a longer half-life (16 hours), thus it can stay in the human body for a longer time [25].

Bone Turnover Markers

sRANKL and CTXI were used as bone resorption markers and OPG and PINP were used as bone formation markers. RANKL is primarily produced by osteoblasts. The binding of RANKL to its receptor in osteoclast accelerates bone resorption. OPG is a receptor that neutralizes RANKL and prevents its interaction with RANK. Such binding is responsible for a decrease in osteoclasts activity [1]. RANKL/OPG ratio is another calculated variable, with higher ratios meaning higher bone resorption. PINP is a peptide byproduct derived by the action of proteases on the N-terminal of type I collagen. CTXI is another peptide derived by the action of cathepsin-K at C-terminal of type I collagen [17]. According to the International Osteoporosis Foundation (IOF) and the International Federation of Clinical Chemistry (IFCC), both PINP and CTXI were recommended to be used as bone formation and resorption markers, respectively [16].

Laboratory analysis

Biological Specimen (Serum)

Blood specimens were collected from the median antecubital vein in two 10 mL sterile plain tubes using vacutainer technique. Each specimen was processed within 30 minutes of collection time. This processing includes centrifugation of sterile plain tubes for 15 minutes at 3,000 rpm, then serum was aliquoted in 0.5 mL sterile microtubes. All microtubes were labeled appropriately with the research-coded number and were stored under -80°C until the time of analysis.

Enzyme-linked Immunosorbent Assay (ELISA)

ELISA kits provided by manufacture (MyBioSource, Inc., San Diego, CA) were used for measuring serum sRANKL, OPG, PINP, CTXI, and cotinine. Manufacturer guidelines for sample processing, measurement, and readings were followed. Serum samples that were previously stored at -80°C were used directly after thawing at room temperature. In our study, we used a sandwich ELISA. The detection range of exposure marker (cotinine) was 0.5-16 ng/mL. The detection ranges for bone formation markers were 0.156-10 ng/mL and 15.63-1000 pg/mL for OPG and PINP, respectively. The detection ranges for bone resorption markers were 15.625-1000 pg/mL and 0.16-10 ng/mL for sRANKL and CTXI, respectively.

Serum samples were diluted 1/8, 1/10, 1/20, and 1/200 for sRANKL, cotinine, CTXI, and PINP, respectively. Undiluted serum samples were used for serum OPG. The concentration of bone turnover markers and cotinine in each sample was determined based on the dilution factor and calculation compared the average sample readings at 450 nm with the readings of the standard curve. In our study, all marker levels were reported as ng/mL. Intra-assay CV in our laboratory were 3.8%, 5.8%, 5.3%, 3.7%, and 3.9% respectively for OPG, PINP, sRANKL, CTXI, and cotinine. Manufacturer-reported intra-assay CV were <10%, 5.1%, 10%, 6.2%, and <15% for OPG, PINP, sRANKL, CTXI, and cotinine, respectively.

Sample size estimation and statistical analysis

Sample Size Estimation

The sample size was calculated for analysis of covariance (ANCOVA) to see if there were any differences in the adjusted mean [Least Square Means (LSM)] of the outcomes variables if we control for the effect of potential confounders: age, BMI, physical activity, milk/dairy and alcohol intake. The G* Power 3.1.9.2 was used to calculate sample size based on the following parameters: effect size (Cohen’s f) = 0.35 (medium effect), two-sided test, alpha = 0.05, power = 0.80, number of group = 2, number of covariates = 5; the required total sample size should be 68 (34/group). The actual sample size in our study was 73, 35 were smokers and 38 were nonsmokers.

Statistical Analysis

IBM SPSS version 24 (IBM Corp., Armonk, NY) was used to analyze the data. All collected data were evaluated using descriptive statistics to examine the distribution of data values, including outliers and patterns of missing values. The differences in characteristics between two groups of smokers and nonsmokers were analyzed using independent t-test or the Mann-Whitney U test for continuous data, and Chi-squared or Fisher's exact test for categorical data.

To detect for differences in the adjusted means (LSM) for levels of bone turnover markers between smokers and nonsmokers, data were analyzed using an ANCOVA to adjust for effect of covariates: age, BMI, physical activity, milk/dairy intake, and alcohol intake. Criteria of including covariate in ANCOVA analyses were based on the theoretical model that explained the effect of smoking on bone turnover. The ANCOVA was evaluated for assumptions of normality, and homogeneity of variances. ANCOVA test is known to be robust for violation of normality assumptions with attention to type I error, and it is conditionally robust for violation of homogeneity of variance. In our study, the violation of the assumption of normality was remediated through Box-Cox (-1/Y) inverse transformations. Also, ANCOVA analysis was evaluated for an assumption to the homogeneity of regression slopes. If homogeneity of regression slope was violated due to the interaction between smoking and covariate, then the analysis would have been modeled for such effect by fitting non-parallel slopes to each group. All hypotheses were tested as two-sided at a significance level of p-value ≤ 0.05 and 95% confidence intervals.

## Results

Sample characteristics

Demographics

Our study enrolled 73 participants, 35 smokers and 38 nonsmokers. The mean age of 73 participants was 34.84 ± 6.51 years and mean BMI was 27.12 ± 3.52. The mean for age and BMI were similar in both groups. Both groups were similar concerning race, marital status, education, working status, and income distributions. Of all participants, the majority were white (n = 51, 70%), married (n = 33, 45%), had college or two-year degree (n = 24, 33%), working for pay (n = 58, 79%) with an annual income of $25,000-$50,000 (n = 29, 40%). Most smokers resided in a suburban area (n = 20, 57%), while the largest proportion of nonsmokers were rural (n = 17, 45%) (Table 1).

Lifestyle and Clinical Characteristics

Table 1 includes details about participant’s lifestyles and clinical characteristics that may influence serum bone turnover markers. The groups were similar in terms of self-reported significant changes in body weight, use of vitamin/mineral supplements, exposure to secondhand smoke, and levels of weight-bearing exercise and alcohol intake. However, the groups differed in terms of physical activity and milk/dairy intake. The average for performing physical activities was lower among smokers than nonsmokers (2.23 ± 1.78 vs. 2.95 ± 1.47, p = 0.02). Milk and dairy intake were lower among smokers than nonsmokers (1.71 ± 1.47 vs. 3.00 ± 1.87, p = 0.001).

**Table 1:** Sample characteristics Values are presented as mean (standard deviation) or number (%). BMI: Body mass index.

Variables	All Participants (n = 73)	Smokers (n = 35)	Nonsmokers (n = 38)	p-value
Age, yr	34.84 (6.74)	34.06 (6.97)	35.55 (6.05)	0.39
Race/Ethnicity				0.65
Black/African American (non-Hispanic)	18 (24.7)	10 (28.6)	8 (21.1)	
Asian/Pacific Islanders	1 (1.4)	1 (2.8)	0 (0)	
Latino or Hispanic	3 (4.1)	1 (2.8)	2 (5.3)	
White/Caucasian (non-Hispanic)	51 (69.8)	23 (65.7)	28 (73.7)	
Marital status				0.70
Married	33 (45.2)	15 (42.8)	18 (47.3)	
Living with the partner	11 (15.1)	5 (14.3)	6 (15.8)	
Divorced	3 (4.1)	1 (2.8)	2 (5.3)	
Married, but separated	2 (2.7)	2 (5.7)	0 (0)	
Never married	24 (32.9)	12 (34.3)	12 (31.6)	
Education level				0.07
Some high school, but did not graduate	5 (6.8)	2 (5.7)	3 (7.9)	
High school graduate	18 (24.7)	9 (25.7)	9 (23.7)	
Some college or two-year degree	24 (32.9)	12 (34.3)	12 (31.6)	
Four-year college graduate	16 (21.9)	11 (31.4)	5 (13.1)	
More than a four-year college degree	10 (13.7)	1 (2.8)	9 (23.7)	
Living environment				0.01
Rural	22 (30.2)	5 (14.3)	17 (44.7)	
Suburban	32 (43.8)	20 (57.1)	12 (31.6)	
Urban	19 (26.0)	10 (28.6)	9 (23.7)	
Living with other individuals				0.28
Yes	60 (82.2)	27 (77.1)	33 (86.9)	
No	13 (17.8)	8 (22.9)	5 (13.1)	
Annual household/income				0.26
Below $25,000	24 (32.9)	12 (34.3)	12 (31.6)	
$25,000–$50,000	29 (39.7)	13 (37.1)	16 (42.1)	
$51,000–$75,000	14 (19.2)	9 (25.7)	5 (13.1)	
$76,000–$100,000	2 (2.7)	1 (2.8)	1 (2.7)	
Above $100,000	4 (5.5)	0 (0)	4 (10.5)	
Working status				0.06
Working for pay	58 (79.4)	26 (74.3)	32 (84.1)	
Unemployed and looking for work	8 (11.0)	5 (14.3)	3 (7.9)	
Temporarily laid off or on leave	1 (1.4)	0 (0)	1 (2.7)	
Student	4 (5.5)	4 (11.4)	0 (0)	
Other	2 (2.7)	0 (0)	2 (5.3)	
BMI, kg/m^2^	27.12 (3.52)	26.94 (3.59)	27.29 (3.50)	0.68
Significant changes in body weight				0.11
Yes	3 (4.1)	3 (8.6)	0 (0)	
No	70 (95.9)	32 (91.4)	38 (100)	
The number of days of performing the physical activity	2.60 (1.66)	2.23 (1.78)	2.95 (1.47)	0.02*
0	3 (4.1)	3 (8.6)	0 (0)	
1	21 (28.8)	13 (37.1)	8 (21.1)	
2	13 (17.8)	6 (17.1)	7 (18.4)	
3	16 (21.9)	7 (20)	9 (23.7)	
4	13 (17.8)	3 (8.6)	10 (26.3)	
5	1 (1.4)	0 (0)	1 (2.6)	
6	4 (5.5)	1 (2.8)	3 (7.9)	
7	2 (2.7)	2 (5.7)	0 (0)	
Number of days of performing weight-bearing exercise	0.79 (1.50)	0.51 (0.98)	1.05 (1.83)	0.38
0	52 (71.2)	26 (74.2)	26 (68.4)	
1	4 (5.5)	3 (8.6)	1 (2.6)	
2	7 (9.6)	3 (8.6)	4 (10.5)	
3	6 (8.2)	3 (8.6)	3 (7.9)	
5	2 (2.7)	0 (0)	2 (5.3)	
6	2 (2.7)	0 (0)	2 (5.3)	
Taking vitamin/mineral supplements				0.64
Yes	13 (17.8)	7 (20)	6 (15.8)	
No	60 (82.2)	28 (80)	32 (84.2)	
The number of days of milk/dairy intake	2.38 (1.80)	1.71 (1.47)	3 (1.87)	0.001
0	10 (13.7)	6 (17.1)	4 (10.5)	
1	16 (21.9)	11 (31.5)	5 (13.1)	
2	17 (23.3)	12 (34.3)	5 (13.1)	
3	15 (20.5)	3 (8.6)	12 (31.6)	
4	2 (2.7)	1 (2.8)	1 (2.6)	
5	10 (13.7)	1 (2.8)	9 (23.7)	
7	3 (4.1)	1 (2.8)	2 (5.3)	
Average for number of daily alcohol consumption	0.70 (1.08)	0.86 (1.14)	0.55 (1.01)	0.187
0	45 (61.6)	19 (54.2)	26 (68.4)	
1	14 (19.2)	7 (20)	7 (18.4)	
2	7 (9.6)	5 (14.3)	2 (5.3)	
3	5 (6.8)	3 (8.6)	2 (5.3)	
4	2 (2.7)	1 (2.8)	1 (2.6)	
5 & above	0 (0)	0 (0)	0 (0)	
Exposure to indoor secondhand smoke				0.25
Yes	7 (9.6)	5 (14.3)	2 (5.3)	
No	66 (90.4)	30 (85.7)	36 (94.7)	
Duration of smoking, yr		5.89 (3.16)		
The primary mode of smoking				
Manufactured cigarettes		35 (100)		
Number of smoked cigarettes/days		10.14 (6.36)		
Number of smoked cigarettes/weeks		70.46 (43.77)		

Smoking Status

Table 1 shows the smoking history of smokers. All these smokers used manufactured cigarettes as a primary mode of smoking. The average number of cigarettes smoked per day was 10.14 ± 6.36 and 70.46 ± 6.36 cigarettes a week. The median EXCO was higher in smokers than nonsmokers (13 vs. 4 ppm, range: 6-36 vs. 2-5, p < 0.001). Also, the median serum cotinine was higher in smokers than that of nonsmokers (79.6 vs. 3.56 ng/mL, range: 29.17-208.64 vs. 0.48-19.32 ng/mL, p < 0.001) (Table 2).

**Table 2:** Comparison of tobacco exposure markers between smokers and nonsmokers Values are presented as median, range, and interquartile range Q1-Q3. EXCO: Exhaled carbon monoxide.

Variable	Smokers (n = 35)	Nonsmokers (n = 38)	Z	p-value
EXCO, ppm	13, 6-36, (8-18)	4, 2-5, (3-5)	-7.38	<0.001
Serum cotinine, ng/mL	79.21, 29.17-208.64, (54.23-128.60)	3.56, 0.48-19.32, (2.28-4.85)	-7.34	<0.001

Differences in levels of bone turnover markers

Due to the inability to randomize groups and effects that selected behavioral and dietary variables can have on bone metabolism, an ANCOVA was used to statistically control for confounding related to group differences in age, BMI, physical activity, milk/dairy intake, and alcohol intake. In terms of OPG and PINP bone formation markers, least-square means (LSM - means adjusted for covariates) were similar for smokers and nonsmokers. For OPG, LSM was 1.54 ± 0.30 ng/mL for smokers, and 1.66 ± 0.31 ng/mL for nonsmokers [F (1,66) = 2.40, p = 0.126]. PINP LSM was 56.44 ± 7.51 ng/mL for smokers, and 58.77 ± 7.45 ng/mL for nonsmokers [F (1,66) = 1.62, p = 0.208] (Table 3). Results were different for bone resorption markers, as LSM were different for smokers and nonsmokers for CTXI, with LSM for smokers lower than that for nonsmokers [0.82 ± 0.83 vs. 1.30 ± 0.82 ng/mL, F (1,66) = 5.73, p = 0.020] (Table 3).

**Table 3:** ANCOVA results: adjusted means for serum bone markers by smoking status while controlling for age, BMI, physical activity, milk dairy, and alcohol intake LSM: Least square mean; *η_p_*^2^: partial eta square; *η*^2^: eta square; OPG: Osteoprotegerin; PINP: Procollagen type I N-terminal propeptide; CTXI: Carboxyl-terminal telopeptide of collagen type I.

			Main Effect: Smoking			Overall Model		
Variables	Smokers LSM (SD) (n = 35)	Nonsmokers LSM (SD) (n = 38)	F (1, 66)	p-value	η_p_^2^	F (6, 66)	p-value	η^2^
OPG, ng/mL	1.54 (0.30)	1.66 (0.31)	2.40	0.126	0.035	0.92	0.489	0.077
PINP, ng/mL	56.44 (7.51)	58.77 (7.45)	1.62	0.208	0.024	0.38	0.889	0.033
CTXI, ng/mL	0.82 (0.83)	1.30 (0.82)	5.73	0.020	0.080	2.27	0.047	0.171

Levels of both sRANKL and RANKL/OPG ratio were a lack in conformance with our statistical model assumption of normality in distribution; therefore, such violation was remediated using Box-Cox (-1/Y) inverse transformations. Results for the inverse transformed sRANKL and RANKL/OPG ratio appear in Tables 4-6 and Tables 7-9, respectively. For sRANKL, the main effect for smoking [F (1,64) = 10.74, p = 0.002] and the smoking by physical activity interaction [F (2,64) = 8.63, p = 0.001] were significant. In addition, the smoking by alcohol intake interaction approached significance [F (2,64) = 3.1, p = 0.052] (Table 4). Finding of smoking by alcohol interaction was exceeding limits for maintaining of type I error rate and this may be due to underpowered ANCOVA analyses; such findings need further validation in larger studies. As those interactions indicate that the covariate effect (slope) differs depending on smoking status, simple main effects analyses were performed, and the resulting parameter estimates controlling for smoking status are presented in Table 5. There was an independent linear association between physical activity and sRANKL for smokers [β = 0.259, t = 4.150, p < 0.001], but not for nonsmokers [β = -0.012, t = -0.18, p < 0.858]. Simple main effects analysis for the smoking by alcohol interaction found an independent linear association between alcohol intake and smoking status for smokers [β = 0.212, t = 2.191, p = 0.032], but not for nonsmokers [β = -0.117, t = -1.129, p = 0.263]. Table 6 shows that the LSM of transformed sRANKL was statistically significantly lower in smokers than nonsmokers [1.64 ± 0.60 vs. 1.69 ± 0.62 ng/mL, F (1,64) = 10.74, p = 0.002]. Special attention should be given to the LSM of transformed data, higher LSM should be interpreted as lower mean and vice versa.

**Table 4:** ANCOVA results for serum sRANKL (transformed): Model of separate slope analysis (fitting non-parallel slopes) sRANKL: soluble-receptor activator of nuclear factor-kappa B ligand;* η_p_*^2^: partial eta square;* η*^2^: eta square (used only for overall model).

Variables	df	F	p-value	η_p_^2 ^/ η^2 ^
Smoking Status	(1, 64)	10.74	0.002	0.144
Age, yr	(1, 64)	0.11	0.747	0.002
BMI, kg/m^2^	(1, 64)	0.00	0.990	0.000
Milk/Dairy Intake	(1, 64)	1.15	0.287	0.018
Smoking Status & Physical Activity	(2, 64)	8.63	<0.001	0.212
Smoking Status & Alcohol Intake	(2, 64)	3.10	0.052	0.088
Overall model	(8, 64)	2.62	0.015	0.247

**Table 5:** ANCOVA results (parameter estimates) for serum sRANKL (transformed): Model of separate slope analysis (fitting non-parallel slopes) sRANKL: soluble-receptor activator of nuclear factor-kappa B ligand;* η_p_*^2^: partial eta square.

Variables	β	t	p-value	η_p_^2^
Smoking Status (Yes)	-0.984	-3.227	0.002	0.144
Age, yr	0.004	0.324	0.747	0.002
BMI, kg/m^2^	0.000	-0.012	0.990	0.000
Milk/Dairy Intake	-0.045	-1.073	0.287	0.018
Smoking Status (Yes) & Physical Activity	0.259	4.150	<0.001	0.212
Smoking Status (No) & Physical Activity	-0.012	-0.180	0.858	0.001
Smoking Status (Yes) & Alcohol Intake	0.212	2.191	0.032	0.070
Smoking Status (No) & Alcohol Intake	-0.117	-1.129	0.263	0.020

**Table 6:** Means of serum sRANKL (transformed) after modeling of non-parallel slopes Values are presented as least square mean (standard deviation). sRANKL: soluble-receptor activator of nuclear factor-kappa B ligand.

Variables	Smokers (n = 35)	Nonsmokers (n = 38)
sRANKL, ng/mL	1.64 (0.60)	1.69 (0.62)

Results of the ANCOVA model for transformed RANKL/OPG ratio produced statistically significant main effect for smoking [F (1,65) = 5.88, p = 0.018] and the smoking by physical activity interaction [F (2,65) = 5.49, p = 0.006] (see Table 7). Results of the simple main effects analysis indicated that there was a statistically significant linear association between physical activity and transformed RANKL/OPG for smokers [β = 0.362, t = 3.305, p < 0.002], but not for nonsmokers [β = -0.051, t = -0.434, p = 0.666] (see Table 8). It is essential to mention that due to the reciprocal transformation for the sRANKL and RANKL/OPG ratio, the sign of parameter estimate (β) should be interpreted opposite to the usual (e.g., a positive coefficient indicates an inverse relationship for the raw value). Table 9 shows that the LSM of transformed RANKL/OPG ratio was lower in smokers than nonsmokers [2.62 ± 1.09 vs. 2.81 ± 1.10 ng/mL, F (1,65) = 5.88, p = 0.018]. Special attention should be given to the LSM of transformed data, higher LSM should be interpreted as lower mean and vice versa.

**Table 7:** ANCOVA results for serum RANKL/OPG ratio (transformed): Model of separate slope analysis (fitting non-parallel slopes) RANKL/OPG ratio: a ratio calculated by dividing soluble-receptor activator of nuclear factor-kappa B ligand on osteoprotegerin; *η_p_*^2^: partial eta square; *η*^2^: eta square (used only for overall model).

Variables	df	F	p-value	η_p_^2^/ η^2^
Smoking Status	(1, 65)	5.88	0.018	0.083
Age, yr	(1, 65)	0.92	0.341	0.014
BMI, kg/m^2^	(1, 65)	0.05	0.821	0.001
Milk/Dairy Intake	(1, 65)	0.18	0.674	0.003
Alcohol Intake	(1, 65)	0.13	0.716	0.002
Smoking Status & Physical Activity	(2, 65)	5.49	0.006	0.145
Overall model	(7, 65)	1.82	0.099	0.164

**Table 8:** ANCOVA results (parameter estimates) for serum RANKL/OPG ratio (transformed): Model of separate slope analysis (fitting non-parallel slopes) RANKL/OPG ratio: a ratio calculated by dividing soluble-receptor activator of nuclear factor-kappa B ligand on osteoprotegerin; *η_p_*^2^: partial eta square.

Variables	β	t	p-value	η_p_^2^
Smoking Status (Yes)	-1.260	-2.425	0.018	0.083
Age, yr	0.020	0.958	0.341	0.014
BMI, kg/m^2^	-0.008	-0.227	0.821	0.001
Milk/Dairy Intake	-0.032	-0.423	0.674	0.003
Alcohol Intake	0.047	0.366	0.716	0.002
Smoking Status (Yes) & Physical Activity	0.362	3.305	0.002	0.144
Smoking Status (No) & Physical Activity	-0.051	-0.434	0.666	0.003

**Table 9:** Means of serum RANKL/OPG ratio (transformed) after modeling of non-parallel slopes Values are presented as least square mean (standard deviation). RANKL/OPG ratio: a ratio calculated by dividing soluble-receptor activator of nuclear factor-kappa B ligand on osteoprotegerin.

Variables	Smokers (n = 35)	Nonsmokers (n = 38)
RANKL/OPG ratio, ng/mL	2.62 (1.09)	2.81 (1.10)

## Discussion

There is increasing evidence in support of the inverse effect of tobacco smoking on bone mass as demonstrated by lower BMD in tobacco smokers, therefore, it can be inferred that tobacco smoking would also influence bone turnover markers. Our study was conducted to test that hypothesis. Concerning bone formation markers, the first marker was the OPG. Our study found adjusted mean serum OPG levels were lower in smokers than that of nonsmokers; however, such a difference was not significant. Such findings of non-significant differences in adjusted means of serum OPG levels between smoker and nonsmoker were found to be in concordance with a study conducted in patients with periodontitis [23]. Meanwhile, three studies that compared smokers with nonsmokers in healthy middle-aged adult males [20], and patients from both sexes with chronic periodontitis [21,22], reported that smokers had significantly lower OPG levels than nonsmokers. However, Mizrak et al. reported that Albino rats exposed to nicotine delivered in their water had significantly higher mean plasma OPG levels than that of controls [19]. Contrary findings in Albino rats should be carefully interpreted since rats were exposed only to nicotine and not a large number of compounds contained in tobacco smoke. Those other compounds (e.g., aryl hydrocarbons) provide additional pathways for tobacco smoke to affect both osteoclasts and osteoblasts. Pure nicotine has been found to have a nonlinear dose-response by osteoblasts: in low doses, osteoblast activity was increased, while high doses decreased osteoblast production, resulting in cell death [26]. Our finding is consistent with published results that found bone formation inversely affected by smoking; however, non-significant finding for the differences in the adjusted means of serum OPG levels may be potentially explained by the small sample size and/or by adding covariates which account for very little variance in the dependent variable (OPG levels) that might actually reduce power; such findings need further validation in larger studies and open the door to investigate for other covariates that may account for large variance in the dependent variable.

PINP is another bone formation marker that was measured in our study. Our study found that the adjusted mean serum PINP levels were similar in smokers and nonsmokers. PINP is a newly recommended marker for bone formation, so it has not been widely reported in the literature. The most commonly investigated bone formation marker in the literature is osteocalcin (OC). Compared with those studies, our findings were concordant with three studies conducted in healthy adult males [12,20,27], and with the Gao et al. study where Wistar rats were exposed to tobacco secondhand smoke [18]. Meanwhile, one study conducted in patients with periodontitis found that the mean saliva OC levels were significantly lower in smokers than nonsmokers [24].

We examined two bone resorption markers in this study, namely CTXI and sRANKL, along with a ratio of bone resorption to bone formation (RANKL/OPG ratio). The adjusted mean serum CTXI levels were found to be lower in smokers compared to nonsmokers. Kargin et al. reported similar findings for lower serum CTXI levels in smokers than nonsmokers (p = 0.007) [20]. Neither Kargin et al. nor our findings were consistent with the hypothesis that bone resorption markers are higher in smokers. In another study conducted in healthy adults, Khoja et al. reported that mean serum CTXI levels were similar in smokers and nonsmokers [27].

Several studies have examined bone resorption markers other than CTXI. Two studies conducted in healthy adults reported that smokers had higher, though not statistically significant, mean plasma TRACP-5b [12,28], and lower, but not statistically significant, mean urinary N-terminal telopeptide (u-NTX) [28]. Besides, one study of patients with periodontitis reported that smokers had higher, but not statistically significant, mean saliva C-telopeptide Pyridinoline Cross-links of Type I (ICTP) than nonsmokers [24]. Finally, a study by Gao et al. found Wistar rats exposed to secondhand smoke had higher levels for mean serum TRACP-5b than that of controls who were not exposed to secondhand smoke [18].

Another bone resorption marker measured in our study was sRANKL. In an ANCOVA model that adjusting for selected covariates which affect bone metabolism, the main effects of smoking and the interaction (smoking and physical activity) were found to be statistically significant. Meanwhile, the effect of interaction (smoking and alcohol intake) was close to the statistical significance level. The interpretation of the simple main effect of these interactions should be reversed due to inverse transformation. For smokers, higher physical activity led to lower sRANKL levels; however, for nonsmokers higher physical activity had no effect on the sRANKL levels. Also, for smokers, higher ingestion of alcohol led to lower sRANKL levels, but for nonsmokers, higher ingestion of alcohol had no effect on the sRANKL levels. Kargin et al. reported lower mean serum RANKL levels in healthy smokers than that of nonsmokers, but such a difference was not statistically significant [20]. Also, three studies that enrolled patients with periodontitis reported that there was no significant difference in mean RANKL levels between smokers and nonsmokers [21-23]. None of those studies adjusted for relevant covariates. Kargin et al. advanced the potential explanation that some of the nonsmoker subjects may have been exposed to secondhand smoke, which was not measured in their study, unlike in this study, in which secondhand smoking was measured and found to be similar in both groups. Furthermore, this study was adjusted for covariates and excluded any diseases or conditions that might influence serum sRANKL.

Concerning tobacco smoking and other lifestyles, Cusano reported that smokers usually consume more alcohol, perform less physical activities, and consume less dietary calcium than nonsmokers [6]. Concerning the combined behavior of smoking and alcohol intake, Kim et al. reported that blood total alkaline phosphatase (ALP) activity was significantly lower in participants who had alcohol drinking and smoking than that of the control group (p < 0.05) [29].

RANKL/OPG ratio is another variable that reflects the balance between bone formation and resorption. RANKL/OPG ratio was reported to be increased in diseases or pathological conditions associated with high bone resorption such as multiple myeloma. The interpretation of the simple main effect of smoking by physical activity interaction effect should be reversed due to inverse transformation. For smokers, higher physical activity led to lower levels of RANKL/OPG ratio, while for nonsmokers, higher physical activity had no effect on the RANKL/OPG ratio. In a study conducted in healthy participants, Kargin et al. reported that smokers had a higher but not statistically significant mean RANKL/OPG ratio than that of nonsmokers [20]. In three studies conducted in patients with periodontitis, two reported higher mean RANKL/OPG ratio in smokers than nonsmokers [21,22], while one reported higher, but not statistically significant mean RANKL/OPG ratio in smokers than nonsmokers [23]. None of those studies adjusted for covariate effects. Our findings support the hypothesis that bone resorption is increased in smokers as evidenced by a higher level of sRANKL and RANKL/OPG ratio. However, CTXI was lower in smokers, which requires further investigation.

The earlier paragraphs discussed differences in means for each bone turnover marker between groups of smokers and nonsmokers. On one hand, the overall findings indicate the LSM of bone formation markers were lower in smokers than that of nonsmokers; however, such differences were not found to be statistically significantly differed between the two groups. These findings are consistent with our hypothesis that smoking decreased bone formation activity and maybe the lack of power is responsible for nonsignificant differences. On the other hand, the LSM of bone resorption markers were found to be statistically significantly differed between smokers and nonsmokers. The LSM for sRANKL and RANKL/OPG ratio were found to be higher in smokers than nonsmokers; however, the LSM for CTXI was lower in smokers than nonsmokers. These findings were consistent with our hypothesis that smoking increased bone resorption activity; however, the CTXI finding was not consistent with our hypothesis but it was similar to findings reported by Kargin et al.. Further large studies were needed to explain these findings.

There are several strengths in this study. First, clear and strict eligibility criteria were used to recruit participants which may minimize the effect of other confounders on bone turnover markers. Second, this study is one of few studies investigating relationships between smoking and bone turnover markers in participants free from periodontitis. Third, we measured more than one marker for bone formation and resorption markers that were recommended by IOF and IFCC. Fourth, this study utilized both EXCO and serum cotinine as an exposure marker for tobacco smoking along with a self-reported questionnaire. Besides, this study is one of the few studies to adjust for relevant confounders.

The following limitations were identified for this study: The cross-sectional design of this study limits the attribution of causation for smoking on serum levels of bone turnover markers. The quantitative nutritional assessment for participant's intake of calcium was not evaluated. Such measurement may act as covariate accounts for the large variance in the dependent variable; therefore, it may enhance the finding of the ANCOVA model. The measurements for certain hormones and vitamins that may influence bone turnover markers were not carried out; however, all participants were screened and were excluded if they had any of disorders that were associated with the imbalances of these hormones and vitamins. Also, any participants who were in uses of these hormones or vitamins were excluded from our study. Despite that, it is possible that some participants may have an abnormal level of these vitamins and hormones but were not diagnosed with these disorders which may bias our finding of bone turnover markers. Finally, the diagnostic test for bone mass was not carried out in our study. Such a diagnostic test acts as another indicator for imbalances in bone turnover and may strengthen our findings.

Based on our findings, we can say tobacco smoking influences bone turnover markers, particularly those involved with resorption. Our findings were consistent with other studies, with small differences potentially due to variation in age, sample size, the intensity of smoking, and the adjustment for covariates. Also, earlier studies enrolled participants from both sexes and/or recruited only patients with periodontitis. Furthermore, some of the earlier studies measured bone turnover markers in fluid other than serum (e.g., saliva and urine). It seems that the relationships between smoking and bone turnover markers are quite complex and most studies examining such relationships used a cross-sectional design, so additional, large-scale longitudinal studies are required to have a comprehensive understanding for effect of smoking on bone turnover markers.

## Conclusions

Our study provides evidence for the effect of smoking on bone resorption markers in the young adult male population. Such an effect should be carefully considered side by side with other poor healthy lifestyles that may lead to poor bone health and increase the risk for osteoporosis. Information about the adverse effects of smoking on bone health should be included in programs to prevent smoking or encourage smoking cessation.
